# Distinct effects of rosuvastatin and rosuvastatin/ezetimibe on senescence markers of CD8+ T cells in patients with type 2 diabetes mellitus: a randomized controlled trial

**DOI:** 10.3389/fendo.2024.1336357

**Published:** 2024-03-22

**Authors:** Sang-Hyeon Ju, Joung Youl Lim, Minchul Song, Ji Min Kim, Yea Eun Kang, Hyon-Seung Yi, Kyong Hye Joung, Ju Hee Lee, Hyun Jin Kim, Bon Jeong Ku

**Affiliations:** ^1^ Division of Endocrinology and Metabolism, Department of Internal Medicine, Chungnam National University Hospital, Daejeon, Republic of Korea; ^2^ Division of Endocrinology and Metabolism, Department of Internal Medicine, Chungnam National University Sejong Hospital, Sejong, Republic of Korea; ^3^ Department of Internal Medicine, Chungnam National University School of Medicine, Daejeon, Republic of Korea; ^4^ Department of Medical Science, Chungnam National University School of Medicine, Daejeon, Republic of Korea

**Keywords:** diabetes mellitus, Type 2, Hydroxymethylglutaryl-CoA reductase inhibitors, ezetimibe, CD8-Positive T-Lymphocytes, cellular senescence

## Abstract

**Objectives:**

Chronic low-grade inflammation is widely recognized as a pathophysiological defect contributing to β-cell failure in type 2 diabetes mellitus (T2DM). Statin therapy is known to ameliorate CD8+ T cell senescence, a mediator of chronic inflammation. However, the additional immunomodulatory roles of ezetimibe are not fully understood. Therefore, we investigated the effect of statin or statin/ezetimibe combination treatment on T cell senescence markers.

**Methods:**

In this two-group parallel and randomized controlled trial, we enrolled 149 patients with T2DM whose low-density lipoprotein cholesterol (LDL-C) was 100 mg/dL or higher. Patients were randomly assigned to either the rosuvastatin group (N=74) or the rosuvastatin/ezetimibe group (N=75). The immunophenotype of peripheral blood mononuclear cells and metabolic profiles were analyzed using samples from baseline and post-12 weeks of medication.

**Results:**

The fractions of CD8+CD57+ (senescent CD8+ T cells) and CD4+FoxP3+ (T_reg_) significantly decreased after intervention in the rosuvastatin/ezetimibe group (−4.5 ± 14.1% and −1.2 ± 2.3%, respectively), while these fractions showed minimal change in the rosuvastatin group (2.8 ± 9.4% and 1.4 ± 1.5%, respectively). The degree of LDL-C reduction was correlated with an improvement in HbA1c (R=0.193, *p*=0.021). Changes in the CD8+CD57+ fraction positively correlated with patient age (R=0.538, *p*=0.026). Notably, the fraction change in senescent CD8+ T cells showed no significant relationship with changes in either HbA1c (*p*=0.314) or LDL-C (*p*=0.592). Finally, the ratio of naïve to memory CD8+ T cells increased in the rosuvastatin/ezetimibe group (*p*=0.011), but not in the rosuvastatin group (*p*=0.339).

**Conclusions:**

We observed a reduction in senescent CD8+ T cells and an increase in the ratio of naive to memory CD8+ T cells with rosuvastatin/ezetimibe treatment. Our results demonstrate the immunomodulatory roles of ezetimibe in combination with statins, independent of improvements in lipid or HbA1c levels.

## 1 Introduction

Type 2 diabetes mellitus (T2DM) is a chronic metabolic disorder, with alterations in the immune system contributing to both the disease’s onset and the progression of its complications, including cardiovascular disease (CVD) (1, 2). In South Korea, hypercholesterolemia, a prominent risk factor for CVD, is found in 83.3% of T2DM patients, of which 26.9% are receiving treatment for this condition (3, 4). Of those receiving lipid-lowering therapy, statin monotherapy and the statin/ezetimibe combination are the predominant treatments for hypercholesterolemia, accounting for 73.9% and 15.6%, respectively (5). Given the high rate of treatment with these agents, understanding the beneficial effects of statin and statin/ezetimibe, such as their immunomodulatory roles, will be crucial for optimizing therapeutic strategies and enhancing patient outcomes.

Statins act as selective and competitive antagonists of the 3-hydroxy-3-methylglutaryl coenzyme-A (HMG-CoA) reductase, thereby inhibiting cholesterol biosynthesis. In addition, statins exhibit beneficial pleiotropic effects, including improvement in endothelial function, inhibition of vascular inflammation, reduction of oxidative stress, and plaque stabilization (6–10). Most importantly, statins have demonstrated anti-inflammatory and immunomodulatory properties across various autoimmune diseases (11, 12). The mechanisms underlying their immunologic effects involve the induction and tissue migration of T_reg_ and the inhibition of Th1 and Th17 differentiation (13). On the other hand, ezetimibe inhibits the absorption of cholesterol by inhibiting intestinal cholesterol transporter NPC1L1, and its combination with statins achieves lipid-lowering goals more effectively than statin monotherapy (14). Ezetimibe has been reported to reduce CD4+ memory T cells in cardiac transplant recipients and to promote differentiation into anti-inflammatory M2 macrophages rather than pro-inflammatory M1 macrophages (15). Given the distinct and overlapping immunomodulatory effects of both statins and ezetimibe, a study on the influence of combined statin/ezetimibe on immune cell subsets is required.

Chronic inflammation is now recognized as a key factor in the onset of T2DM and its associated complications (2). This inflammation is driven by cytokines released from both activated and senescent immune cells (16–19). The senescence of CD8+ T cells contributes to persistent, low-grade inflammation via the secretion of pro-inflammatory cytokines and the production of cytotoxic molecules like granzyme B and perforin (20, 21). In T2DM patients, dysfunction of CD8+ T cells (22, 23) and accumulation of senescent CD8+ T cells (24) have been reported. Moreover, the senescent CD8+ T cells in T2DM are related to the development of cardiovascular complications (25, 26). Therefore, based on the immunomodulatory roles of statin and ezetimibe, investigating the impact of these drugs on the senescence of CD8+ T cells is warranted.

In terms of lipid-lowering efficacy, the combination of statin and ezetimibe therapy has demonstrated an additive effect, with each exerting their own mechanisms. We hypothesized that the combination therapy may enhance immunomodulatory efficacy beyond that of statin monotherapy, particularly regarding the senescence of CD8+ T cells. Additionally, we aim to ascertain if these effects correlate with their lipid-lowering properties. These insights could guide more tailored therapeutic approaches and enhance the efficacy of treatment for comorbidities of T2DM.

## 2 Patients and methods

### 2.1 Trial design and eligibility

We conducted a two-group parallel, single-center, randomized controlled trial (RCT) to compare the effects of a combination of rosuvastatin and ezetimibe (experimental group) with rosuvastatin alone (control group). A total of 149 patients were randomized into the experimental (N=75) and control (N=74) groups.

We enrolled patients with T2DM and LDL-C levels of 100 mg/dL or higher from the Diabetes Center of Chungnam National University Hospital, a tertiary teaching hospital. Detailed eligibility criteria are as follows:

*Inclusion Criteria*

1. Males or females aged > 18
2. Diagnosis of T2DM as per the 2022 American Diabetes Association’s Standards of Medical Care
3. LDL-C levels ≥ 100 mg/dL

*Exclusion Criteria*

1. Current use of lipid-lowering therapy, including statins, ezetimibe, fibrates, nicotinic acid, bile acid sequestrants, and PCSK9 inhibitors
2. Postmenopausal hormone replacement therapy
3. Aspartate aminotransferase (AST) or alanine aminotransferase (ALT) levels > 2.5-fold of the upper normal limit
4. Creatine kinase (CK) levels > 3-fold of the upper normal limit
5. Malignant tumor diagnosis or treatment within the past 5 years
6. Poorly controlled or uncontrolled hypothyroidism (thyroid-stimulating hormone (TSH) > 1.5-fold of the upper normal limit)
7. Known hypersensitivity to the drugs used in the study: rosuvastatin, ezetimibe, and their combination.

Recent guidelines recommend targeting an LDL-C level of less than 100 mg/dL in patients with diabetes of 10 years or shorter duration who lack cardiovascular risk factors, and advocate for a more stringent LDL-C goal in those with cardiovascular risk factors (4, 27). Accordingly, this study selected patients in need of dyslipidemia management with LDL-C levels of 100 mg/dL or higher. Patients with conditions or receiving treatments known to affect lipid metabolism, such as postmenopausal hormone replacement therapy, cancer therapies, and hypothyroidism, were excluded (28, 29). Patients who already had elevated liver enzymes or creatine kinase levels, which are indicators of adverse events of study drug were also excluded.

### 2.2 Ethics statement

This research adhered to the Declaration of Helsinki and the Ethical Guidelines for Clinical Research. The study complied with both the Standard Protocol Items: Recommendations for Interventional Trials (SPIRIT) 2013 statement (30) and the revised CONSORT guideline (31). The Ethics Committee of the Institutional Review Board (IRB) of Chungnam National University Hospital approved this study on November 8, 2018 (IRB file No. CNUH 2018-10-030). All participants provided written informed consent and retained the right to withdraw or discontinue participation at any time. Our protocol was registered on clinical research information service (CRIS; KCT0003477).

### 2.3 Interventional process

**Figure 1:**
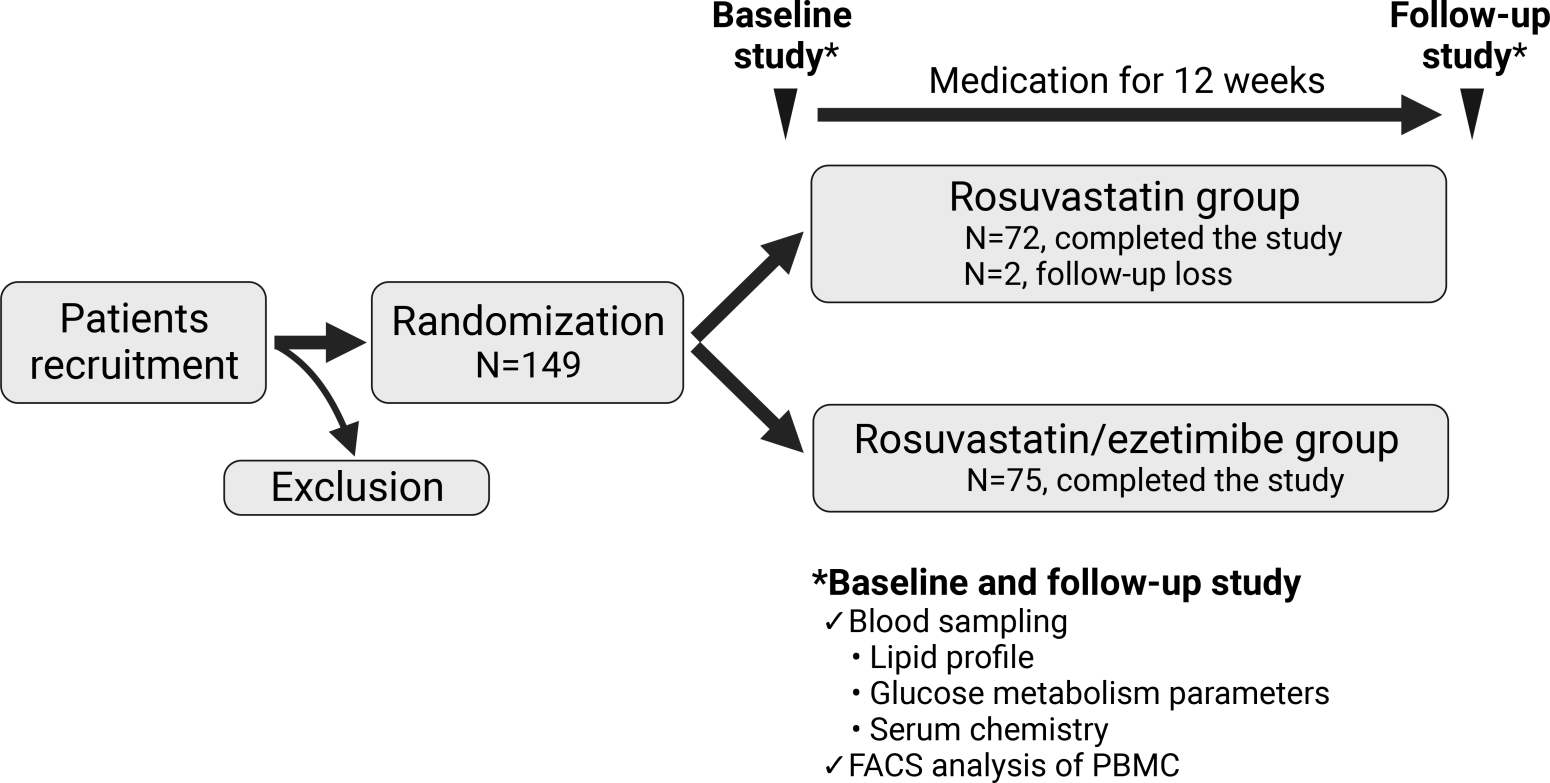
Scheme of the study design. The flow chart represents a prospective, double-blind, randomized, single-center study with 149 participants. Participants were randomized into two groups: one receiving rosuvastatin alone (N=74), and the other receiving a combination of rosuvastatin and ezetimibe (N=75). Assessments were conducted at both baseline and after 12 weeks of medication for all participants. FACS, fluorescence activated cell sorting; PBMC, peripheral blood mononuclear cell.

Enrolled patients were assigned to either the rosuvastatin/ezetimibe group or the rosuvastatin-alone group. All participants underwent routine diabetes care over the 12-week study period. The combination group received rosuvastatin 5mg with ezetimibe 10mg, while the control group received rosuvastatin 5mg per day for 12 weeks of intervention. Participants took their medication 30 minutes post-breakfast daily. Blood samples were drawn at the start and end of the study to assess lipid and glycemic parameters, serum chemistry, and surface markers of peripheral blood mononuclear cells (PBMCs) (**Figure 1**).

### 2.4 Randomization and masking

Random allocation sequences were created using SPSS Version 26.0. Participants were blinded, receiving a number from the doctor to obtain medication at the pharmacy. Post data collection at 12 weeks, the blind was lifted.

### 2.5 Outcome measurements

*Primary Outcome*

1. Proportion of CD8+ T cells with senescence markers (CD28-CD57+CD8+ T cells)

*Secondary Outcomes*

1. Proportion of CD4+ T cells with senescence markers (CD28-CD57+CD4+ T cells)
2. Lipid profile, including triglyceride, total cholesterol, HDL-C, and LDL-C
3. Glycemic parameters, including HbA1c, fasting glucose, and HOMA indices
4. Serum chemistry, including blood urea nitrogen (BUN), creatinine, estimated glomerular filtration rate (eGFR), and liver enzymes (AST and ALT)

### 2.6 Laboratory and analytical procedures

At the beginning and conclusion of the study, peripheral blood samples were drawn into serum separator tubes (SST). LDL-C levels were quantified using the Cobas 6000 analyzer (Roche Diagnostics, Basel, Switzerland) through enzymatic colorimetric techniques.

For the analysis of PBMCs, we followed procedures based on a previously published article by our group (32). The study utilized several monoclonal antibodies, including anti-CD4-AF700, anti-CD8-PE, anti-CD8-APC, anti-CD28-APC, anti-CD57-FITC, anti-interferon (IFN)-γ-PE-Cy7, and anti-tumor necrosis factor (TNF)-α-APC, IL-17A-APC, Granzyme B-PE, perforin-APC, all sourced from eBioscience, San Diego, CA, USA. Following permeabilization, cells were stained for intracellular cytokines and cytotoxic molecules using anti-IFN-γ-PE-Cy7 and anti-TNF-α-APC. Multicolor flow cytometry was performed with the BD LSRFortessa flow cytometer (BD Biosciences, San Jose, CA, USA). Data analysis was conducted using FlowJo V10 software (FlowJo, LLC, Ashland, OR, USA). Blood samples with a PBMC viability of 80% or more were used for FACS analysis, with 77 patients meeting this criterion at both baseline and follow-up visits.

### 2.7 Safety evaluation

All adverse events were documented, emphasizing potential side effects of the intervention drugs: muscle pain/weakness, headache/dizziness, sleep problems, and digestive symptoms including nausea, indigestion, diarrhea, and constipation. Serum chemistry tests for AST, ALT, and CK were employed to identify asymptomatic adverse drug effects.

### 2.8 Statistical analysis

Baseline and 12-week data were collected on standardized datasheets, anonymizing patient identity. Numerical data are presented as mean ± standard deviation (SD), while discrete data are shown as numbers with their corresponding percentages (%). Intra-group differences were evaluated using paired t-tests, and inter-group differences using unpaired t-tests. Results were considered statistically significant if *p* < 0.05. Analyses were performed using SPSS Version 26.0 (IBM Corp., Armonk, NY, USA), with graphical representations created using GraphPad Prism 9.4.1 (GraphPad Software Inc., San Diego, CA, USA) and OriginPro 2021 (OriginLab Corp., Northampton, MA, USA).

## 3 Results

### 3.1 Baseline characteristics

In this RCT, we enrolled 149 patients diagnosed with T2DM whose low-density lipoprotein cholesterol (LDL-C) was 100 mg/dL or higher. They were randomized into either the rosuvastatin or the rosuvastatin/ezetimibe group, with both groups taking the medication for 12 weeks (**Figure 1**). Among the total patients enrolled, 147 patients (N=72 in the rosuvastatin group and N=75 in the rosuvastatin/ezetimibe group) completed blood sampling at both baseline and follow-up. Baseline metrics, including age, sex ratio, body mass index (BMI), duration of T2DM, and biochemical parameters reflecting their lipid and glucose metabolism, kidney function, and inflammation markers, did not exhibit statistically significant differences between the two groups (**Table 1**).

**Table 1:** Baseline characteristics.

	Rosuvastatin	Rosuvastatin/Ezetimibe	
Parameters	N=74	N=75	*p* value
Age (years)	56.9 ± 11.1	53.6 ± 12.4	0.091
Sex, male	33 (44.6%)	35 (46.7%)	0.464
BMI (kg/m^2^)	25.2 ± 4.6	26.0 ± 4.1	0.250
T2DM duration (years)	6.2 ± 7.1	4.9 ± 6.4	0.233
HbA1c (%)	7.2 ± 1.6	7.0 ± 1.4	0.287
Insulin (μIU/ml)	14.38 ± 8.82	18.20 ± 14.28	0.103
C-peptide (ng/ml)	1.52 ± 0.60	1.74 ± 1.16	0.614
HOMA-IR	5.8 ± 3.7	7.2 ± 6.4	0.200
HOMA-β (%)	92.6 ± 96.3	89.5 ± 72.5	0.860
Triglyceride (mg/dL)	165.8 ± 91.7	178.0 ± 97.7	0.435
Total cholesterol (mg/dL)	213.9 ± 26.5	213.5 ± 32.7	0.936
HDL-C (mg/dL)	51.8 ± 13.2	48.5 ± 12.3	0.116
LDL-C (mg/dL)	137.4 ± 23.4	136.1 ± 23.2	0.739
BUN (mg/dL)	16.1 ± 4.6	15.1 ± 4.2	0.166
Creatinine (mg/dL)	0.72 ± 0.19	0.72 ± 0.18	0.946
eGFR (mL/min/1.732m^2^)	98.3 ± 16.2	99.2 ± 19.1	0.749
hsCRP (mg/dL)	2.1 ± 3.8	1.8 ± 2.3	0.581

BMI, body mass index; T2DM, type 2 diabetes mellitus; HOMA-IR, homeostatic model assessment of insulin resistance; HOMA-β, homeostatic model assessment of beta cell function; HDL-C, high-density lipoprotein cholesterol; LDL-C, low-density lipoprotein cholesterol; BUN, blood urea nitrogen; eGFR, estimated glomerular filtration rate; hsCRP, high-sensitivity C-reactive protein. Data are presented as mean ± SD or number (corresponding proportion in percentage).

### 3.2 Effect of rosuvastatin and rosuvastatin/ezetimibe on T cell senescence

**Figure 2:**
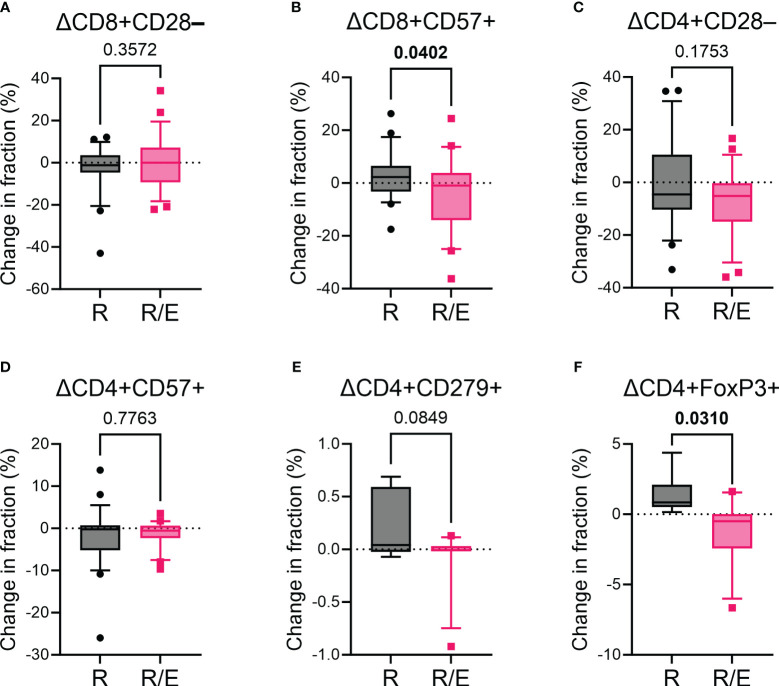
Impact of rosuvastatin and rosuvastatin/ezetimibe on markers for senescence and exhaustion of T cells. **(A, B)** Changes in the fraction of CD8+CD28– **(A)** and CD8+CD57+ **(B)** senescent CD8+ T cells in the rosuvastatin (R) and rosuvastatin/ezetimibe (R/E) groups. **(C, D)** Changes in the fraction of CD4+CD28– **(C)** and CD4+CD57+ **(D)** senescent CD4+ T cells in the rosuvastatin (R) and rosuvastatin/ezetimibe (R/E) groups. **(E, F)** Changes in the fraction of CD4+CD279+ **(E)** and CD4+FoxP3+ **(F)** CD4+ T cells in the rosuvastatin (R) and rosuvastatin/ezetimibe (R/E) groups. Data are presented in box-and-whisker plots, where the whiskers represent the 10th to 90th percentiles. Unpaired t-tests were used.

We analyzed the surface markers of PBMCs at baseline and after a 12-week intervention to evaluate T cell subsets (**Figure 1**). We evaluated markers of T cell senescence (loss of CD28 and expression of CD57), exhaustion (expression of PD-1, also known as CD279), and regulatory T cells (expression of FoxP3) (33). Although the change in the proportion of CD8+CD28– T cells during the 12-week intervention was comparable between the rosuvastatin and combination groups, there was an increase in CD8+CD57+ T cells in the rosuvastatin group, while this proportion decreased in the rosuvastatin/ezetimibe group (**Figures 2A, B**). In contrast, the change in the proportion of senescent CD4+ T cells (CD4+CD28– or CD4+CD57+) showed no significant difference between the two groups (**Figures 2C, D**). The change in the proportion of PD-1-expressing CD4+ T cells (CD4+CD279+) did not differ significantly between the two groups (**Figure 2E**). Additionally, regulatory T cells (T_reg_; CD4+FoxP3+) were significantly decreased in the combination group compared to the rosuvastatin group (**Figure 2F**). These results suggest that the two medications have distinct effects on T cell subsets, notably with declines in the proportion of CD8+CD57+ senescent T cells and T_reg_ cells in the rosuvastatin/ezetimibe group.

### 3.3 Lipid and glycemic parameters and their relationship with T cell senescence markers

Following 12 weeks of lipid-lowering medication, levels of triglyceride, total cholesterol, and LDL-C declined in both groups. However, the combined group experienced a more significant reduction in total cholesterol and LDL-C levels (**Supplementary Table 1**). Although changes in HbA1c and HOMA indices remained non-significant, the combined group registered a more pronounced decrease in serum insulin levels (**Supplementary Table 1**).

**Figure 3:**
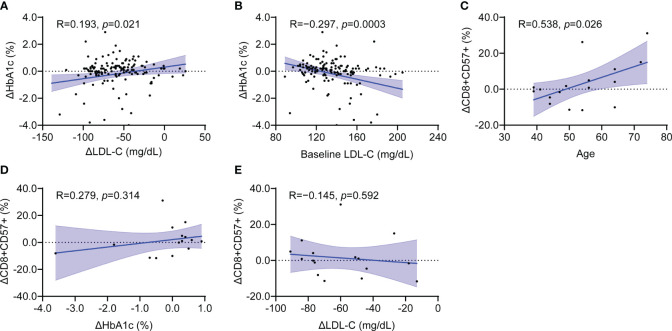
Clinical parameters associated with CD8+CD57+ senescence T cell marker in the total patient group. **(A, B)** Scatter plot showing the relationship between changes in HbA1c and changes in LDL-C **(A)** as well as baseline LDL-C **(B)**. **(C–E)** Scatter plot showing the relationship between changes in CD8+CD57+ T cells with age **(C)**, changes in HbA1c **(D)** and LDL-C **(E)**. LDL-C, low-density lipoprotein cholesterol. Simple linear regression tests were used. The blue shaded area indicates the 95% confidence interval.

To explore potential relationship between immunosenescence and metabolic parameters, we conducted correlation analyses on changes in proportion of senescent CD8+ T cells (ΔCD8+CD57+) and various lipid and glycemic parameters. In the total patient group, the HbA1c change (ΔHbA1c) was positively correlated with the LDL-C change (ΔLDL-C) and negatively correlated with baseline LDL-C (**Figures 3A, B**). Age was positively correlated with ΔCD8+CD57+ T cells (**Figure 3C**). The relationship between ΔLDL-C or LDL-C and ΔHbA1c was maintained in both the rosuvastatin and combination groups (**Supplementary Figures 1A, B, F, G**). The correlation significance between age and ΔCD8+CD57+ T cells diminished in the subgroup analysis (**Supplementary Figures 1C, H**). Interestingly, the ΔCD8+CD57+ T cells did not show a significant correlation with HbA1c or LDL-C alterations, and this finding was consistent in each subgroup analysis (**Figures 3D, E**; **Supplementary Figures 1D, E, I, J**). Meanwhile, the proportion of senescent CD4+ T cells did not correlate significantly with ΔHbA1c but was negatively correlated with ΔLDL-C (**Supplementary Figure 2A, B**). Additionally, the proportion change of CD8+CD28– T cells showed no significant correlation with ΔLDL-C (**Supplementary Figures 2C, D**). Taken together, despite noticeable changes in lipid and glycemic parameters from the combined treatment, there was no significant correlation between CD8+ senescent T cell markers and shifts in HbA1c or LDL-C levels.

### 3.4 Effect of rosuvastatin and rosuvastatin/ezetimibe on the ratio of Naïve to memory T cells

**Figure 4:**
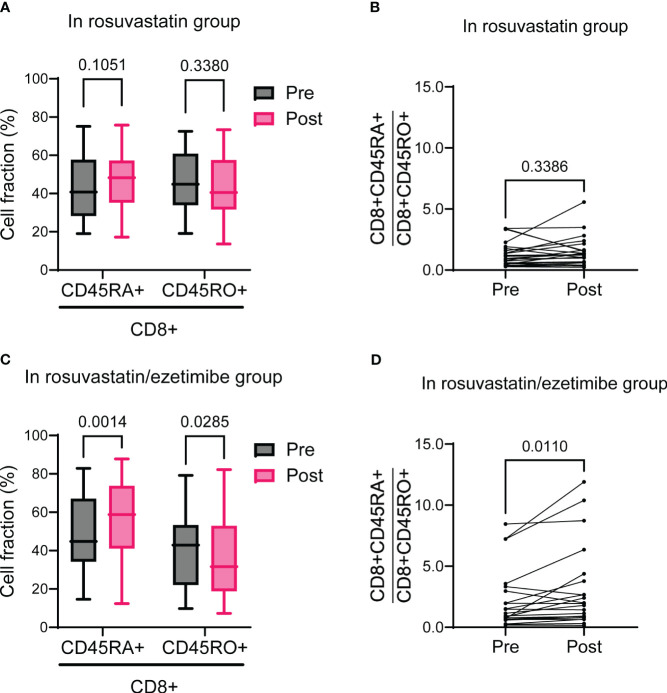
Naïve to memory CD3+CD8+ T cells before and after rosuvastatin or rosuvastatin/ezetimibe treatment. **(A, B)** Pre- and post-intervention fractions of naïve (CD45RA+) and memory (CD45RO+) CD8+ T cells **(A)** and the naïve to memory CD8+ T cell ratio **(B)** in the rosuvastatin group. **(C, D)** Pre- and post-intervention fractions of naïve (CD45RA+) and memory (CD45RO+) CD8+ T cells **(C)** and the naïve to memory CD8+ T cell ratio **(D)** in the rosuvastatin/ezetimibe group. In **(A, C)**, data are presented as Tukey’s box-and-whisker plots, and two-way ANOVA with Šídák’s multiple comparisons tests were used for statistical analysis. In **(B, D)**, paired t-tests were used.

To assess the relative changes in naïve (CD45RA+) versus memory (CD45RO+) T cell populations post-intervention, we analyzed their respective proportions and ratios. In the rosuvastatin group, both the naïve and memory CD8+ T cell proportions remained unchanged, resulting in no significant change in the ratio of naïve to memory CD8+ T cells (**Figures 4A, B**). Notably, in the rosuvastatin/ezetimibe group, the proportion of naïve CD8+ T cells increased while the memory CD8+ T cells decreased, leading to an increased ratio of naïve to memory CD8+ T cells (**Figures 4C, D**). In contrast, the proportions of naïve and memory CD4+ T cells, as well as their ratio, remained unchanged in both the rosuvastatin and combined groups (**Supplementary Figures 3A–D**). Collectively, the combined use of rosuvastatin and ezetimibe shifted the CD8+ T cell balance towards increased naïve proportions without affecting CD4+ T cell subsets.

## 4 Discussion

In this single-center RCT, we aimed to identify the immunomodulatory effects of statin and statin/ezetimibe combination therapy using surface marker analysis of immune cells. Our findings illuminate the distinct impacts these treatments exert on specific T cell subsets. Remarkably, while rosuvastatin monotherapy did not significantly change the proportion of senescent CD8+ T cells, the addition of ezetimibe significantly reduced their proportion. Intriguingly, these observed shifts did not mirror alterations in lipid and glycemic parameters, hinting that the interactions between the medications and T cell senescence are not solely attributed to their lipid-lowering properties. The modulation in the ratio of naïve to memory CD8+ T cells in the combined therapy group further enriches our comprehension, suggesting potential immune rejuvenation, an effect that was not observed in CD4+ T cell subsets. This study demonstrated changes in T cell subsets in response to lipid-lowering therapy, emphasizing the additive effect of ezetimibe in the context of immune dynamics.

Statins exhibit pleiotropic effects via inhibition of the mevalonate pathway, which downregulates nonsteroidal isoprenoid compounds, including geranylgeranyl pyrophosphate (GGPP) and farnesyl pyrophosphate (FPP), downstream mediators of the pathway (13). These mediators are essential for the prenylation of small GTP-binding proteins, including Rho, Rac, and Cdc42, steps pivotal for immune cell activation. Inhibition of prenylation induces SOCS3 expression, promoting T_reg_ differentiation (34). Previous studies indicate increased T_reg_ numbers, tissue infiltration, and Foxp3 expression following statin therapy (13, 35, 36). However, an RCT investigating FoxP3 and IL-10 mRNA expression after 6 months of treatment with statin or statin/ezetimibe found no significant change in FoxP3 expression between LDL-C <70 and ≥70 mg/dL. Moreover, the correlation observed between FoxP3 and IL-10 mRNA expression in the statin group was notably absent in the statin/ezetimibe group, indicating that T_reg_ are not activated (37). In line with these findings, the statin group of our study showed a mild increase in T_reg_ (1.37 ± 1.53%), while a mild decrease (–1.17 ± 2.33%) in T_reg_ was observed in the rosuvastatin/ezetimibe group. In another study, ezetimibe alone reduced the fraction of Th17 cells among PBMCs without affecting that of T_reg_ cells in ankylosing spondylitis patients (38). In rodent study, during a lithogenic high-cholesterol diet, nine months of long-term ezetimibe treatment induced a reduction in T_reg_ cells (39). From these studies, it appears that ezetimibe might either have no effect on, or down-regulate, T cell differentiation into T_reg_ cells. Further studies on the impact of ezetimibe and its combination with statins on T_regs_ are required.

The effects of statin and statin/ezetimibe on CD8+ T cells remain relatively unexplored. Statin therapy reduces CD8+ T cell-mediated inflammatory responses through Kruppel-like factor 2 (KLF2)-dependent mechanisms (40). The LDL receptor, expressed by CD8+ T cells, is crucial for the activation of CD8+ T cells, suggesting that an appropriate concentration of cholesterol is required for CD8+ T cell function (41). In our study, senescent CD8+ T cells (CD8+CD57+) were significantly decreased in the rosuvastatin/ezetimibe group compared to the rosuvastatin group. Notably, senescent CD8+ T cells did not show a significant relationship with HbA1c or LDL-C change, implying that mechanisms other than lipid or glycemic lowering induced the reduction of senescent CD8+ T cells. Previous studies have reported the immune enhancement roles of ezetimibe. In rodent models of liver cancer fed a high-fat high-cholesterol diet, ezetimibe treatment significantly reduced mRNA expression of immune checkpoint genes and immune suppression genes, including *Cd274* (encoding PD-L1), *Cd279* (encoding PD-1), *Ctla4*, *Havcr2*, *Lag3*, and *Entpd2* (42). In another study using syngeneic mouse tumor models, ezetimibe treatment was shown to down-regulate mTOR2 signaling and enhance CPT1A expression, which is associated with the development of CD8+ T cell memory (43, 44). These clues may present potential underlying mechanisms for the additive immunomodulatory effects of ezetimibe. However, further studies are required to elucidate the detailed mechanisms underlying the crosstalk between cholesterol-lowering agents and the immune system, the clinical outcomes dependent on the senescence of CD8+ T cells, and the potential therapeutic implications of combining statins with other immunomodulatory drugs for enhanced patient outcomes.

Hypercholesterolemia, T2DM, and T-cell dysfunction are interrelated. In T2DM patients, there is an expansion of senescent (CD28−CD57+) CD8+ T cells, which are highly inflammatory and secrete cytotoxic mediators while bypassing their antigen specificity (24, 45). T cell dysfunction has now been recognized as a key factor contributing to beta cell failure (2). Furthermore, from our previous study, senescent T cells are associated with systemic inflammation and alter hepatic glucose homeostasis, leading to abnormal glucose homeostasis (32). Conversely, hyperglycemia is recognized as a driver of memory CD8+ T cell dysfunction, rendering patients with T2DM more susceptible to infections (46). These interactions between diabetes and T cell dysfunction ultimately result in chronic inflammation, further exacerbating both conditions. The two-way relationship between T2DM and chronic periodontitis, with senescent CD4+CD28- T cells potentially serving as mediators, is a good example (47). Research from the field of cancer has shown that elevated cholesterol levels induce CD8+ T cell exhaustion, as evidenced by PD-1 expression, through ER stress-XBP1-dependent mechanism. This leads to impaired antitumor function of cytotoxic CD8+ T cells within the tumor microenvironment (48). In line with this, intraosseous simvastatin injection suppressed cancer development by activating cytotoxic CD8+ T cells (49). These findings, along with the adjuvant role of statins in anti-cancer vaccination, supported the inclusion of statins in various anti-cancer trials (50). Considering the intricate connections among hypercholesterolemia, T2DM, and T cell dysfunction, along with their broader implications in conditions such as beta cell failure, infections, chronic inflammation, and even cancer, there is an increasing demand for holistic approaches that include the interplay between the immune system and metabolism. Our study provides foundational insight into these multifaceted interactions, emphasizing the need for integrative therapeutic strategies that concurrently address metabolic imbalances and immune dysregulation.

The study presents previously unexplored results on the effects of statin monotherapy and the combined statin/ezetimibe treatment on T cell subsets and their senescence in patients with T2DM. The strength of this study lies in its RCT design, which included a sizeable patient cohort from a tertiary teaching hospital, ensuring the validity of its findings. Furthermore, the detailed measurements of lipid and glycemic parameters in relation to T cell senescence markers offer a comprehensive overview of lipid-lowering and pleiotropic effects. However, there are some limitations to consider. The study’s single-center nature may limit the generalizability of its results. The relatively short study duration of 12 weeks may not reflect the long-term effects or potential side effects of the medication. Additionally, the study lacks a placebo control group and an ezetimibe-only group, which are necessary to evaluate the individual effects of rosuvastatin, ezetimibe, and combination therapy on the senescence of CD8+ T cells. Although not addressed in this study, future research including non-T2DM patients or patients with other chronic inflammatory conditions may help elucidate potential differences in treatment responses across different patient populations. The follow-up study was conducted only 12 weeks after the initiation of lipid-lowering medication. The lack of lipid profile assessments at multiple timepoints is another limitation. Finally, this study does not investigate the precise molecular mechanisms underlying these effects. While the results provide valuable insights into T2DM treatment, further research is needed to unravel the detailed mechanisms by which these lipid-lowering therapies influence immune cell dynamics.

Based on our findings, future clinical trials should explore the therapeutic potential of statin and ezetimibe therapy through multicenter RCTs with diverse control groups. Our research, utilizing moderate-intensity statin therapy, paves the way for dose optimization studies by varying statin intensity (low, high intensity groups) to assess dose-dependency. Extending the observation beyond our 12-week timeframe to include 6–8 week assessments could provide insights into serial changes by the intervention. Moreover, investigating subgroups based on T2DM duration and cardiovascular risk may uncover the differential immunomodulatory effects of statin and ezetimibe related to disease progression and age. By exploring various doses, treatment durations, and patient subgroups, future clinical trials can build on our results to optimize treatment strategies, improve patient outcomes, and expand the evidence base for clinical decision-making.

## 5 Conclusion

In this RCT, we examined the immunomodulatory effects of statin monotherapy and combined statin/ezetimibe treatment in patients with T2DM. While both treatments demonstrated lipid-lowering properties, the combined statin/ezetimibe treatment notably reduced the proportion of senescent CD8+ T cells, an effect not solely attributed to their lipid-lowering action. Additionally, the combined therapy shifted the balance towards an increased proportion of naive CD8+ T cells, suggesting potential immune rejuvenation. These findings highlight the distinct impacts of statin and ezetimibe on specific T cell subsets, emphasizing the additional immunomodulatory benefit of ezetimibe when combined with statins. Further exploration is warranted to understand the broader implications of these findings for therapeutic strategies in T2DM comorbidities.

## Data availability statement

The raw data supporting the conclusions of this article will be made available by the authors, without undue reservation.

## Ethics statement

This research adhered to the Declaration of Helsinki and the Ethical Guidelines for Clinical Research. The study complied with both the Standard Protocol Items: Recommendations for Interventional Trials (SPIRIT) 2013 statement (30) and the revised CONSORT guideline (31). The Ethics Committee of the Institutional Review Board (IRB) of Chungnam National University Hospital approved this study on November 8, 2018 (IRB file No. CNUH 2018-10-030). All participants provided written informed consent and retained the right to withdraw or discontinue participation at any time. Our protocol was registered on clinical research information service (CRIS; KCT0003477).

## Author contributions

SJ: Writing – original draft, Visualization, Investigation, Formal analysis, Data curation. JoL: Writing – original draft, Visualization, Investigation. MS: Writing – original draft, Visualization, Investigation. JK: Writing – review & editing, Supervision. YK: Writing – review & editing, Supervision. HY: Writing – review & editing, Supervision. KJ: Writing – review & editing, Supervision. JuL: Writing – review & editing, Supervision. HK: Writing – review & editing, Supervision. BK: Writing – review & editing, Supervision, Methodology, Funding acquisition, Conceptualization.
