# Supplementary material for: Distinct effects of rosuvastatin and rosuvastatin/ezetimibe on senescence markers of CD8+ T cells in patients with type 2 diabetes mellitus: a randomized controlled trial
Source: Front Endocrinol (Lausanne). 2024 Mar 22;15:1336357. doi: 10.3389/fendo.2024.1336357 (PMC10996898; doi:10.3389/fendo.2024.1336357)
Supplement: Supplementary file 1 [file DataSheet_1.pdf]

Supplementary Material

Supplementary Figures

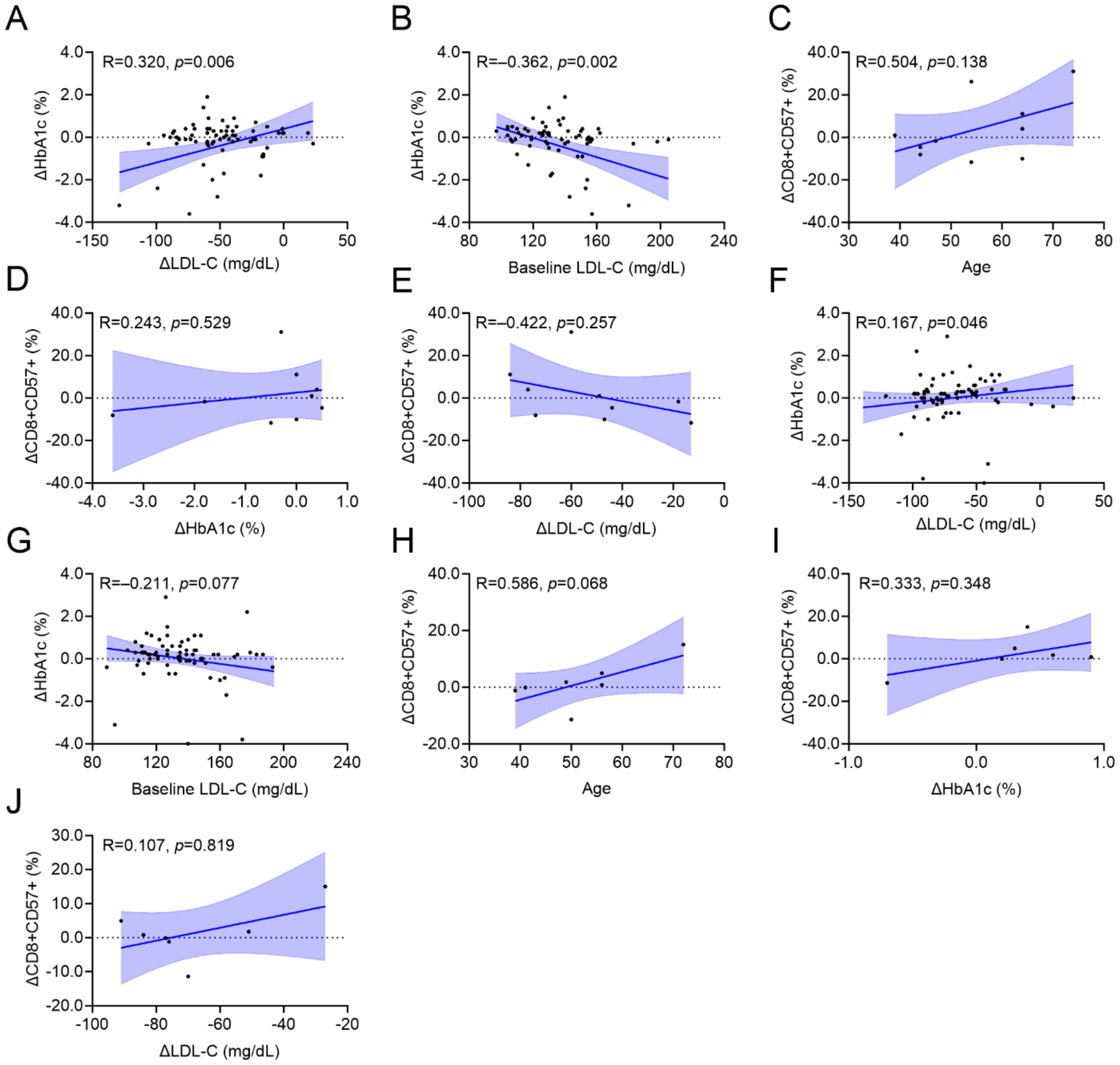

Supplementary Figure 1. Clinical parameters associated with CD8+CD57+, a senescent T cell marker, in the rosuvastatin or rosuvastatin/ezetimibe combination group

(A–B) Scatter plot of the change in HbA1c versus the change in LDL-C (A) and baseline LDL-C (B) in the rosuvastatin group. **(C–E)** Scatter plot of the change in CD8+CD57+ T cells versus age (C), changes in HbA1c (D) and LDL-C (E) in rosuvastatin group. **(F–G)** Scatter plot of the change in HbA1c versus the change in LDL-C (F) and baseline LDL-C (G) in the rosuvastatin/ezetimibe

combination group. **(H–J)** Scatter plot of the change in CD8+CD57+ T cells versus age (H), the changes in HbA1c (I) and LDL-C (J) in the rosuvastatin/ezetimibe combination group. Simple linear regression tests were used. The blue shaded area indicates the 95% confidence interval.

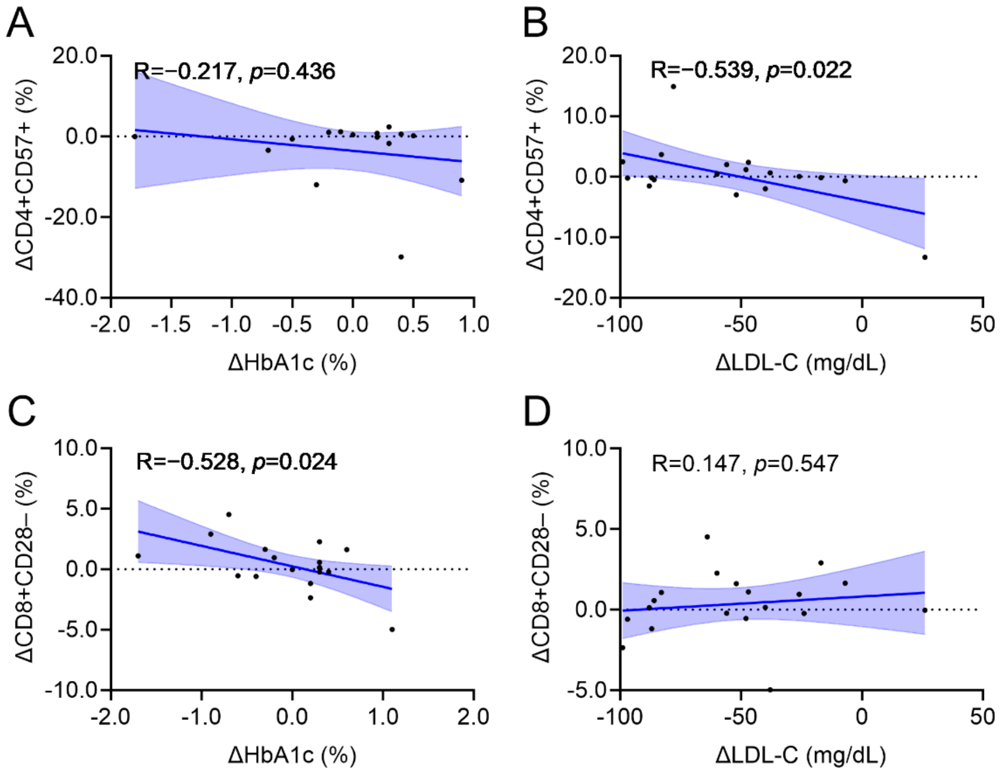

Supplementary Figure 2. Clinical parameters associated with CD4+CD57+ and CD8+CD28– senescence T cell marker.

(A–B) Scatter plot between change in CD4+CD57+ T cell fraction with changes in HbA1c (A) and LDL-C (B). **(C–D)** Scatter plot between change in CD8+CD28– T cell fraction with changes in HbA1c (C) and LDL-C (D). LDL-C, low-density lipoprotein cholesterol. Simple linear regression tests were used. The blue shaded area indicates the 95% confidence interval.

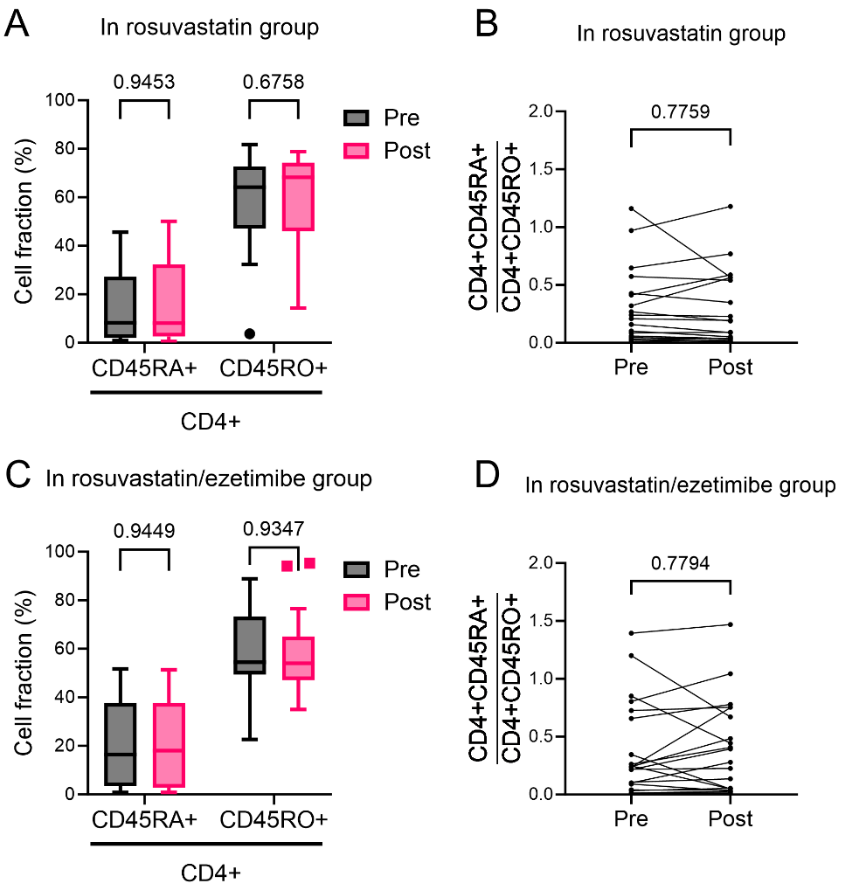

Supplementary Figure 3. Naïve to memory CD3+CD4+ T cells before and after rosuvastatin or rosuvastatin/ezetimibe treatment.

(A–B) Pre- and post-intervention fractions of naïve (CD45RA+) and memory (CD45RO+) CD4+ T cells (A) and naïve to memory CD4+ T cell ratio (B) in the rosuvastatin group. **(C–D)** Pre- and post-intervention fractions of naïve (CD45RA+) and memory (CD45RO+) CD4+ T cells (C) and naïve to memory CD4+ T cell ratio (D) in the rosuvastatin/ezetimibe group. In (A) and (C), data are presented as Tukey's box-and-whisker plots. In (B) and (D), paired t-tests were used.

Supplementary Table

Supplementary Table 1. Impact of rosuvastatin and rosuvastatin/ezetimibe on lipid profile and glucose metabolism parameters.

Parameters	Rosuvastatin<br>N=74	Rosuvastatin/Ezetimibe<br>N=75	*p* value
ΔTriglyceride (mg/dL)	−32.13 ± 84.98	−44.60 ± 85.70	0.379
**ΔTotal cholesterol (mg/dL)**	**−55.51 ± 32.03**	**−76.09 ± 31.35**	**<0.001**
ΔHDL-C (mg/dL)	0.68 ± 7.51	1.86 ± 9.16	0.401
**ΔLDL-C (mg/dL)**	**−50.78 ± 29.85**	**−68.91 ± 27.85**	**<0.001**
ΔHbA1c (%)	−0.41 ± 1.48	0.01 ± 1.15	0.061
**ΔInsulin (μIU/ml)**	**−1.85 ± 10.49**	**−7.80 ± 13.89**	**0.021**
ΔHOMA-IR	−0.60 ± 7.09	−3.23 ± 6.32	0.061
ΔHOMA-β (%)	−20.75 ± 94.93	−33.08 ± 63.19	0.459

HDL-C, high density lipoprotein cholesterol; LDL-C, low density lipoprotein cholesterol; HOMA-IR, homeostatic model assessment of insulin resistance; HOMA-β, homeostatic model assessment of beta cell function. Data are presented as mean ± SD. Values with $p<0.05$ are indicated in bold.
